# GLOBE Observer Data: 2016–2019

**DOI:** 10.1029/2020EA001175

**Published:** 2020-08-05

**Authors:** Helen M. Amos, Matthew J. Starke, Tina M. Rogerson, Marilé Colón Robles, Travis Andersen, Rebecca Boger, Brian A. Campbell, Russanne D. Low, Peder Nelson, David Overoye, Jessica E. Taylor, Kristen L. Weaver, Trena M. Ferrell, Holli Kohl, Theresa G. Schwerin

**Affiliations:** ^1^ NASA Goddard Space Flight Center Greenbelt MD USA; ^2^ Science Systems and Applications, Inc. Lanham MD USA; ^3^ NASA Summer Internship Program Participant, NASA Goddard Space Flight Center Greenbelt MD USA; ^4^ NASA Langley Research Center Hampton VA USA; ^5^ Science Systems and Applications, Inc. Hampton VA USA; ^6^ GLOBE Implementation Office University Corporation for Atmospheric Research Boulder CO USA; ^7^ Earth and Environmental Sciences Brooklyn College Brooklyn NY USA; ^8^ NASA Wallops Flight Facility Wallops Island VA USA; ^9^ Global Science & Technology, Inc. Greenbelt MD USA; ^10^ Institute for Global Environmental Strategies Arlington VA USA; ^11^ College of Earth, Ocean, and Atmospheric Sciences Oregon State University Corvallis OR USA; ^12^ Science Systems and Applications, Inc. Pasadena CA USA

**Keywords:** citizen science, global data set, cloud cover, land cover, tree height, mosquitoes

## Abstract

This technical report summarizes the GLOBE Observer data set from 1 April 2016 to 1 December 2019. GLOBE Observer is an ongoing NASA‐sponsored international citizen science project that is part of the larger Global Learning and Observations to Benefit the Environment (GLOBE) Program, which has been in operation since 1995. GLOBE Observer has the greatest number of participants and geographic coverage of the citizen science projects in the Earth Science Division at NASA. Participants use the GLOBE Observer mobile app (launched in 2016) to collect atmospheric, hydrologic, and terrestrial observations. The app connects participants to satellite observations from Aqua, Terra, CALIPSO, GOES, Himawari, and Meteosat. Thirty‐eight thousand participants have contributed 320,000 observations worldwide, including 1,000,000 georeferenced photographs. It would take an individual more than 13 years to replicate this effort. The GLOBE Observer app has substantially increased the spatial extent and sampling density of GLOBE measurements and more than doubled the number of measurements collected through the GLOBE Program. GLOBE Observer data are publicly available (at observer.globe.gov).

## Key Points

- GLOBE Observer is a mobile app from NASA used worldwide by citizen scientists to collect Earth system observations
- Thirty‐eight thousand participants have submitted 320,000 observations—including 1,000,000 georeferenced photographs—from all seven continents
- It would take an individual more than 13 years to replicate the same sampling effort

## 1 Introduction

The Global Learning and Observations to Benefit the Environment (GLOBE) Program is an international science and education program that launched in 1995 (globe.gov) (Berglund, 1999; Finarelli, 1998; Means, 1998; Muller et al., 2015; Nugent, 2018; Rock et al., 1997). GLOBE Observer is a NASA‐funded citizen science project that is part of the GLOBE Program (observer.globe.gov). GLOBE Observer was founded to help fill data gaps in the GLOBE database and to expand the educational benefits of the GLOBE Program to broader audiences. A 2015 focus group assessment of GLOBE found that while data collection protocols were rigorous, the resulting environmental data were not as widely used in scientific publications as they could be because of widespread temporal and spatial data gaps. Data were primarily collected at schools, during the school day and school year. By accepting environmental observations from any interested volunteer, GLOBE could fill gaps with data collected outside of school grounds and school hours and gather enough data to achieve meaningful geospatial density. GLOBE Observer supports volunteer data collection through a mobile application (“app”), website, training materials, help desk, and other support materials. GLOBE Observer's primary objectives are to (1) increase GLOBE's spatial and temporal data density; (2) enable an increase of scientific and student research; (3) help volunteer users of all ages be part of the GLOBE community; and (4) increase scientific literacy among all participants.

The GLOBE Program offers more than 50 data collection protocols, while GLOBE Observer includes four: clouds, mosquito habitats, land cover, and tree height. These protocols were selected because there was a demand for more of that kind of data from specific science communities and they can be done well by minimally trained volunteers who have no or simple data collection equipment. For each protocol, volunteers are collecting data that would be prohibitively expensive for a science community to collect because of the desired global distribution and repeat timing. For clouds, land cover, and mosquito habitats, data consist of an observation of conditions (cloud cover, mosquito breeding habitat, etc.) along with supporting photographs stamped with location and time that can be used to verify observations. Tree data provide an estimate of tree height with a georeferenced photograph of the tree.

The NASA GLOBE Observer mobile app was launched in 2016 and was created to broaden the opportunities for the general public to contribute to GLOBE as citizen scientists and increase the spatiotemporal density of observations. Through the GLOBE Observer app, participants in GLOBE countries (globe.gov/globe‐community/community‐map) can contribute ground‐based atmospheric, terrestrial, and hydrologic observations complementing NASA's suite of airborne and spaceborne observing platforms. This paper addresses GLOBE Observer's contribution to data density as a foundation for student and science research and serves as a definition of the data collected during the initial years of the project. Previous publications have analyzed subsets of GLOBE Observer data associated with specific sampling events (Aïkpon et al., 2019; Colón Robles et al., 2020; Dodson et al., 2019; Rahman et al., 2019). This paper presents the first summary and analysis of the entire 2016–2019 GLOBE Observer citizen science data set and discusses areas for future improvement.

## 2 GLOBE Observer Mobile App

The GLOBE Observer (GO) app is a NASA‐funded citizen science app available free‐of‐cost in the Apple App Store and Google Play Store (observer.globe.gov/get‐the‐app). The GO app was designed as a tool for the general public (all ages 13+). The app is written in JavaScript, HTML, and CSS, utilizes the AngularJS (2015) framework, and is built using Apache Cordova (2019) for deployment to multiple platforms (i.e., iOS and Android). GO observations are publicly available (at observer.globe.gov). GLOBE has built free on‐line tools to visualize the data on a world map (vis.globe.gov), retrieve data as a comma separated value (csv) file (datasearch.globe.gov), and an application programming interface (API) to facilitate automated or command line data queries (api.globe.gov/search/).

**Figure 1:**
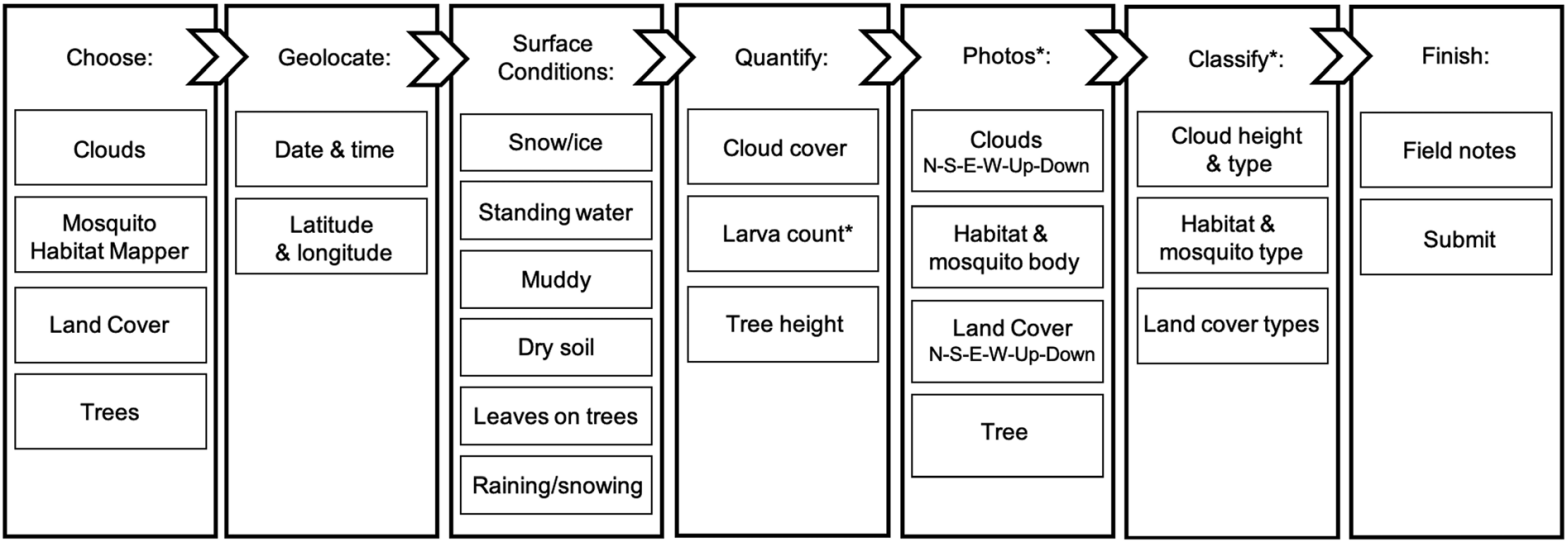
Flowchart of the NASA GLOBE Observer mobile app. Optional elements are indicated by an asterisk. N‐S‐E‐W stands for the four cardinal directions (north, south, east, and west).

Figure 1 shows a flowchart of how the app works and the components of an observation. The GO app currently accepts observations of clouds, mosquito breeding habitats and mosquito type, land cover, and tree heights. There is also a temporary tool for solar eclipses, which was activated in the app for a limited time for the 2017 North American solar eclipse (Dodson et al., 2019) and again for the 2019 South American solar eclipse (GLOBE Observer, 2019). Participants complete required, interactive training in the app to learn how to use the GO tools, after which prompts guide the participant through data collection and submission. For each observation, the internal clock and GPS automatically records date, time, latitude, and longitude. Participants answer a series of six yes/no questions about surface conditions that affect satellite retrievals (e.g., presence of snow or ice on the ground) and then photograph and classify what they see (e.g., cloud type). Numerical estimates, such as number of contrails or number of mosquito larvae, are self‐reported by the participant and manually entered in app. Additional details about the tools in the app are in GLOBE (2019a), and step‐by‐step instructional videos are publicly available (at observer.globe.gov/do‐globe‐observer).

To improve data usability for researchers and to boost science literacy among volunteers, the protocols are aligned with NASA satellite data where feasible. For land cover, participants are shown the land cover classification at their location for the MODIS 250‐m global land cover product (NASA, 2020) and asked to comment on how their observation compares. The clouds protocol is directly linked to daily satellite overpasses. Participants can opt‐in to receive satellite flyover notifications on their device when Aqua (Parkinson, 2003), Terra (Lee et al., 2000), and CALIPSO (Winker et al., 2003) are flying overhead. As encouragement, participants who make a cloud observation within 15 min of a satellite overpass are sent a personalized email containing their cloud observation alongside the satellite observation. The satellite matching is performed by a team at NASA Langley Research Center and also includes matching to geostationary satellites: GOES, Meteosat, and Himawari (Colón Robles et al., 2019). The matching of satellite data to cloud ground observations by citizen scientist started in 1996 with the CERES S'COOL (Student's Cloud Observations On‐Line) Project that compared student cloud type and cover observations with NASA's CERES data (Cloud and the Earth's Radiative Energy System) (Chambers et al., 2017). Once the S'COOL project migrated to be NASA GLOBE Clouds, the team continued to do satellite matches to the CERES instrument. CALIPSO, another project led at NASA Langley Research Center, was recently added. A 5‐year plan is in development to add other satellites and to match satellite data to other GO protocols. Between 1 January 2017 and 1 December 2019, 203,000 GO cloud observations have been matched to satellite observations. GLOBE clouds data with satellite matches for 2017–2019 are publicly available (observer.globe.gov/get‐data/clouds‐data).

## 3 GLOBE Observer Data: 2016–2019

**Figure 2:**
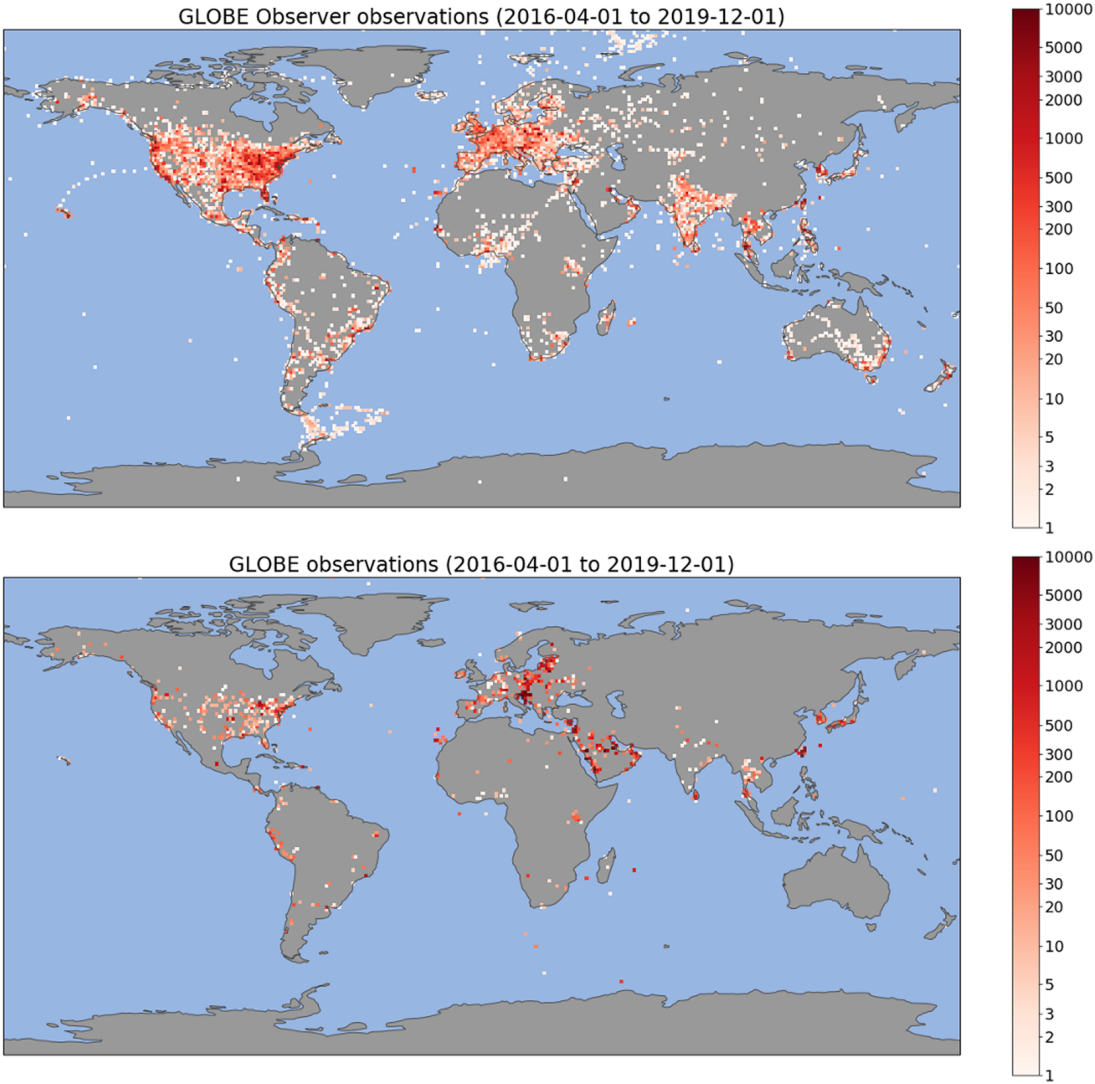
Heatmap of observations submitted via the NASA GLOBE Observer mobile app (top) and traditional channels of GLOBE (bottom) from 1 April 2016 to 1 December 2019. Includes observations from the GLOBE Observer Clouds, Mosquito Habitat Mapper, Land Cover, and Trees tools and equivalent GLOBE protocols.

**Figure 3:**
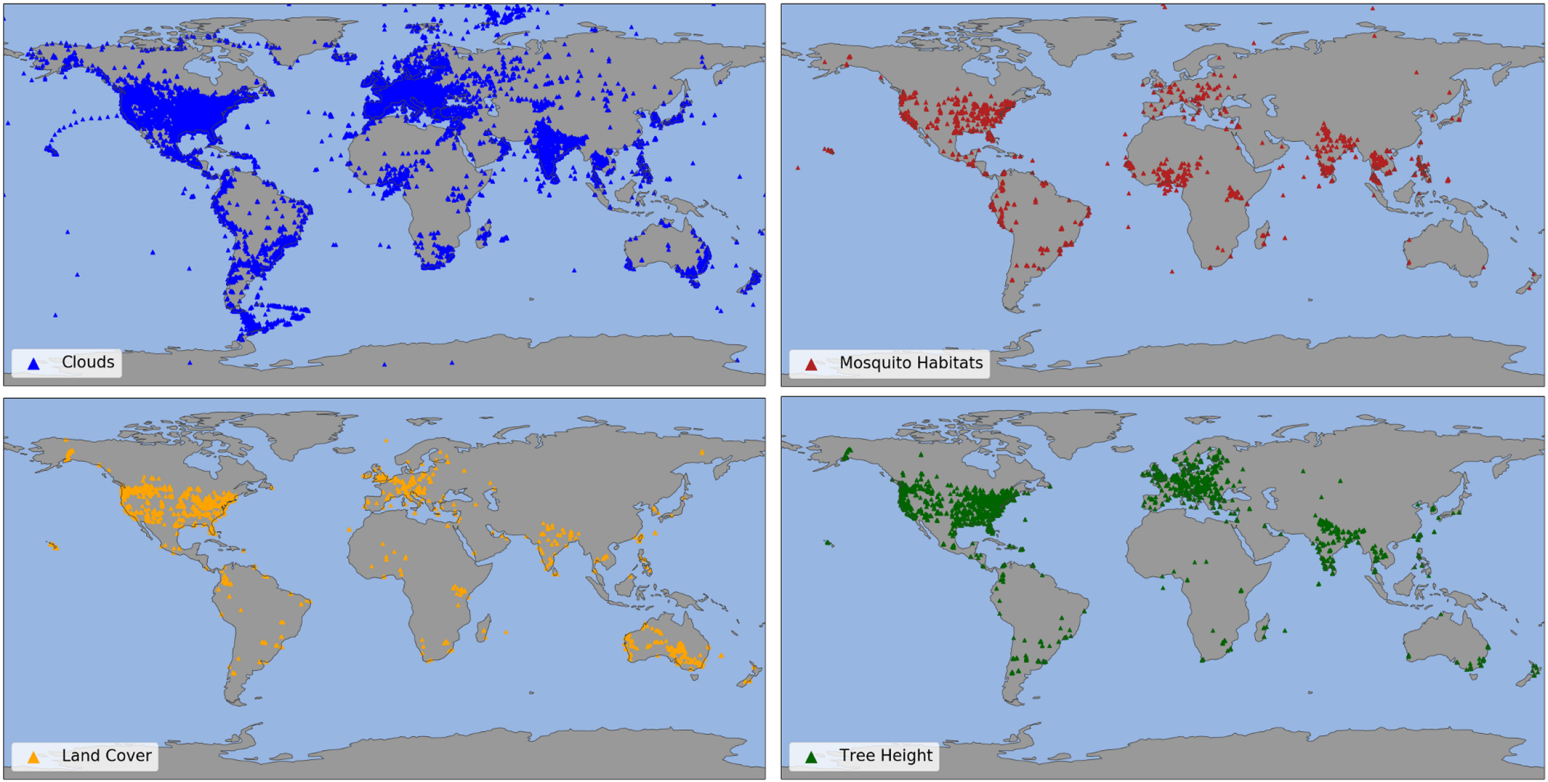
Location of observations made using the NASA GLOBE Observer mobile app from 1 April 2016 to 1 December 2019. Thirty‐eight thousand participants have contributed 320,000 observations worldwide.

Figures 2 and 3 show the geographic coverage of observations made using the app. Thirty‐eight thousand users have submitted 320,000 observations from all seven continents. Assuming (1) it takes a participant 5 min to go outside, find a suitable sampling location, and complete an observation with the GLOBE Observer app and (2) an average work year has 2,000 hr, it would take an individual person 13 work years and 5 months to collect the same number of observations. Assuming a GS‐12 step 1 pay grade for fiscal year 2019 (opm.gov), this would be equivalent to more than $1 million (USD) in salary and does not even include the cost of sampling equipment or the extensive travel it would require to visit the same locations.

The data density is beginning to be great enough to support an increase in scientific and student research, as was one of the original project objectives. From 2017 to 2019, over 500 U.S. student and youth research projects were documented using GLOBE Observer data, which represented an increase from 31 student projects in 2016 (Schwerin & Clark, 2019). The number of scientific and educational publications, articles, and presentations using GLOBE Observer data has also been steadily increasing since 2017 (observer.globe.gov/publications). Peer‐reviewed research applications of GLOBE Observer data include analysis of the 2017 North American solar eclipse (Dodson et al., 2019), analysis of global cloud cover observations collected during a month‐long international data collection blitz (Colón Robles et al., 2020), and mapping the distribution of *Aedes* mosquitoes in west Africa (Aïkpon et al., 2019).

Figure 2 contrasts the spatial distribution of observations submitted through the GLOBE Observer app and through the traditional GLOBE channels. The app has increased the geographic coverage of GLOBE observations. Some geographic gaps in the coverage exist and will persist in countries which are not participating GLOBE countries. Similar to other international citizen science projects, North America and Europe are the most intensely observed regions (e.g., iNaturalist; Chandler et al., 2017). Notable increases in geographic coverage enabled by the app include India, West Africa, South America, and Australia and are discussed below.

Figure 3 shows locations of observations from each app tool individually. The geographic variability in data coverage among the different app tools is multi‐factorial. Each tool was introduced to the app at a different time (Table 1), which accounts for some of the variability. Geographic patterns observed in reported data also reflect the demographics and level of engagement of the GLOBE Program in participating countries prior to a tool's launch in the app. For example, GLOBE schools submitted 87,000 cloud observations in the 12 months prior to the launch of the cloud tool in the app, versus only 1,100 land cover observations submitted in the 12 months prior to the launch of land cover in the app. The difference in activity level seen in traditional GLOBE persists with the app; 35× more cloud observations than land cover observations have been submitted through the app. Another factor driving app activity is internal data collection campaigns, such as the 3‐year (2018–2021) *Trees Around the GLOBE* campaign (GLOBE, 2020). To illustrate this point, the tree height tool in the app has been available for half the time of the land cover tool, but tree heights observations are submitted through the app at twice the rate (1,400 tree height observations per month vs. 650 land cover observations per month, on average). GLOBE also encourages participants to investigate science questions that are meaningful locally. As a result, places where mosquito‐borne diseases are endemic have adopted the mosquito tool more widely. In Figure 3, there are also notable examples on the map of externally driven data collection in India, in Australia by the Australian Scouts, the Arctic, and the Southern Ocean by the Polar Collective (polarcollective.org; Colón Robles et al., 2018). As expected, we see hotspots of data collection around locations where the GLOBE Program and partner organizations have conducted in‐person trainings. In Figure 3, a striking example of the mark of GLOBE trainings are the hotspots of mosquito observations in Thailand (GLOBE, 2019b), Benin (Aïkpon et al., 2019), Peru, and Brazil (mosquito.strategies.org), where extensive trainings took place as a result of initiatives funded by the U.S. Department of State and USAID (Low et al., 2019). GLOBE has also conducted numerous cloud trainings in South America.

**Table 1:** GLOBE Observer Data Summary (1 April 2016 to 1 December 2019)

	Clouds	Mosquito habitats	Land cover	Tree height
Launched in app	April 2016	May 2017	September 2018	March 2019
Number of observations	290,000	19,000	8,400	9,500
Number of observations submitted via traditional GLOBE methods	350,000	1,100^a^	1,500	3,000
Number of photographs	940,000	Habitat: 15,000	45,000	8,200
		Larva: 5,800		
Percent of observations that include at least one photo	77%	Habitat: 80%	95%	86%
		Larva: 30%		
Percent of observations that include 6 of 6 directional photos^b^	41%	n/a	80%	n/a
Percent of observations classified^c^	68%	14%	43%	n/a

*Note*. n/a = not applicable.

^a^ GLOBE formerly supported a pen‐and‐paper version of a mosquito larva observation protocol that is now inactive; 1,100 observations were submitted between 4 October 2015 and 9 August 2019.

^b^ Participants have the option to submit cloud and land cover photos in the four cardinal directions (north, south, east, and west), plus up and down.

^c^ Participants have the option to classify cloud type, mosquito type, and land cover type.

The popularity of the cloud tool in the app compared to the other tools merits further discussion. Cloud observations are more numerous and ubiquitous in part simply because the cloud tool has been in the app longest. Other important contributing factors include the well‐organized volunteer base and outreach materials GLOBE inherited from the NASA S'COOL project, which seeded a community of GLOBE teachers collecting clouds data before the app launched (Chambers et al., 2003, 2017). Due to the resource and staffing demands required to maintain satellite matching in full operation, the cloud tool is currently the only tool within the app that participants can opt‐in to receive notifications of NASA satellite flyovers to their phone and have their satellite matches emailed to them (Colón Robles et al., 2019).

**Figure 4:**
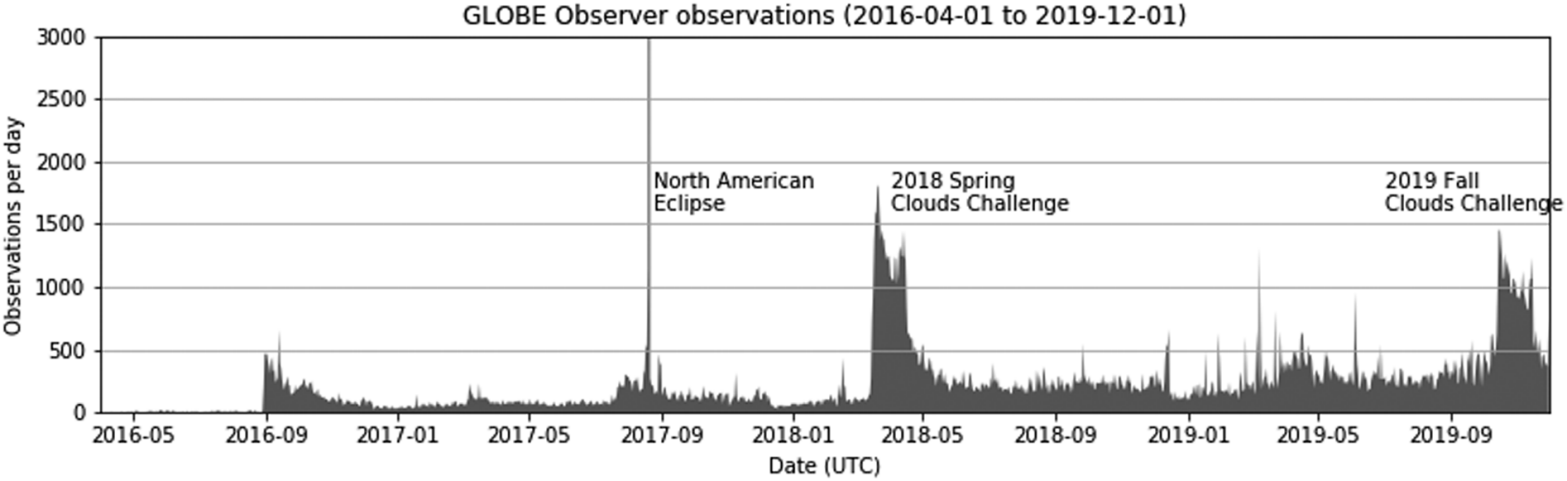
Time series of observations made with the NASA GLOBE Observer mobile app from 1 April 2016 to 1 December 2019. Includes observations from the GLOBE Observer Clouds, Mosquito Habitat Mapper, Land Cover, and Trees tools. The *y*‐axis is cut off at 3,000 observations per day, but on the day of the 2017 North American Total Solar Eclipse more than 18,000 observations were submitted with the GLOBE Observer app.

Figure 4 shows the number of observations submitted per day through the GLOBE Observer app. Large, organized outreach efforts can have a demonstrable impact on data volume and spatiotemporal coverage, such as in the cases of the 2017 North American total solar eclipse (Dodson et al., 2019; Rahman et al., 2019; Weaver et al., 2019) and the 2018 GLOBE Spring Cloud Challenge (Colón Robles et al., 2020; Hayden et al., 2019). Not all outreach efforts produce similar increases in data. A recent example is the 2019 South American solar eclipse (GLOBE Observer, 2019). The absence of a significant effect may be attributable to lack of concurrent NASA promotion (e.g., the 2017 solar eclipse was bolstered by NASA‐wide promotion), geographic domain of the eclipse transit, and historically lower user engagement in the region. By most metrics, the 2018 Cloud Challenge has outperformed all other outreach events and is closely examined in Colón Robles et al. (2020). In 1 month, the 2018 Spring Cloud Challenge brought in 56,000 observations and attracted data submissions from 5,800 new, unique app users. The GLOBE Program as a whole has experienced a sustained increase in daily submissions since. App users during the 2018 Spring Cloud Challenge used the app in a unique way and reported wildfire smoke, extreme haze, and dust storms affecting their areas, with photographs of these phenomena. In 2019, a Fall Cloud Challenge was organized (15 October 2019 to 15 November 2019) promoting sky observations like those unique reports of smoke, haze, and dust storms with a guide on how to enter them on the app (Colón Robles, 2019). The 2019 Fall Cloud Challenge was heavily promoted on social media, but was not picked up by other media (e.g., television stations) like the 2018 Challenge. Even so the 2019 Challenge resulted in over 45,000 observations in 93 countries and attracted data submissions from 2,100 new, unique app users.

**Figure 5:**
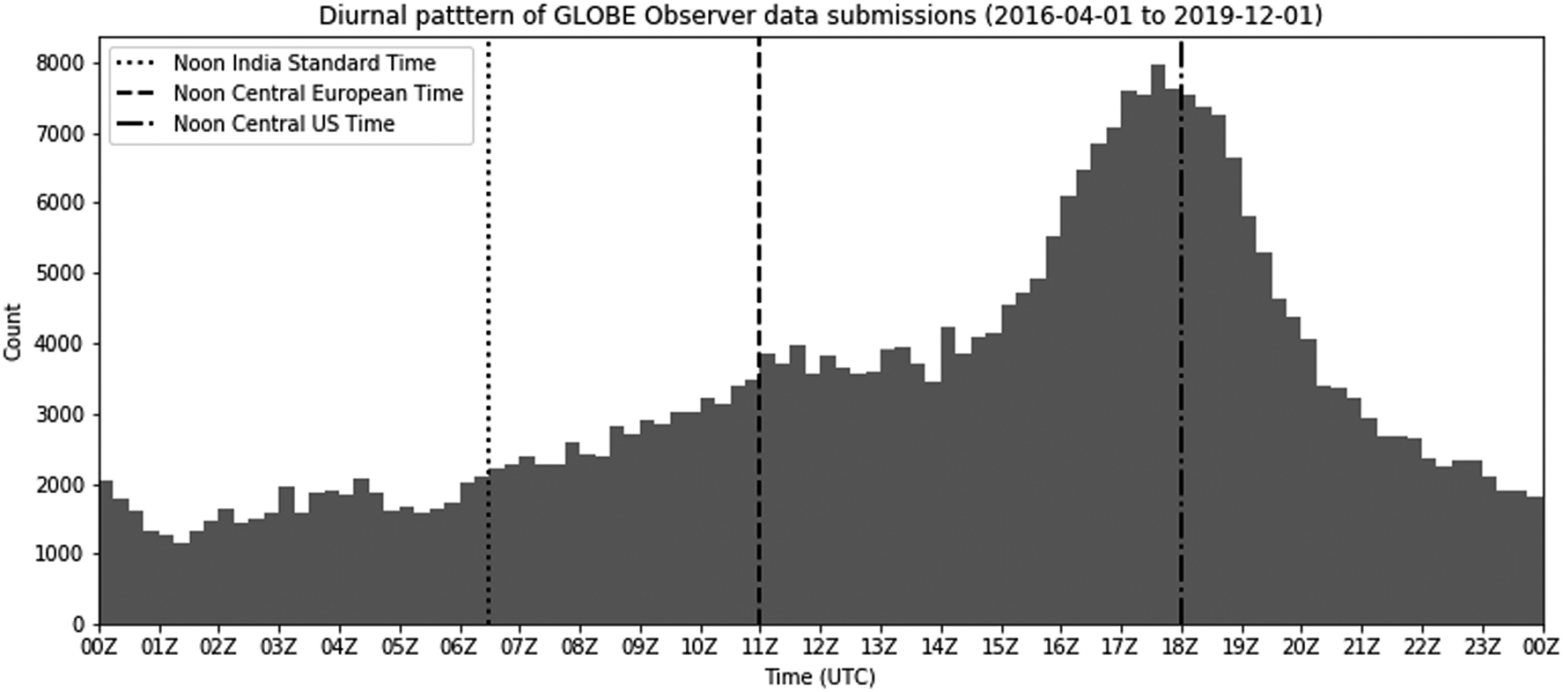
Diurnal frequency of data submissions through the GLOBE Observer mobile app. Includes observations from the GLOBE Observer Clouds, Mosquito Habitat Mapper, Land Cover, and Trees tools during the period 1 April 2016 to 1 December 2019.

Figure 5 shows the diurnal distribution of submissions coming through the GLOBE Observer app. Cloud observation submissions dominate. The peak around 11:00 UTC is driven by participation in Europe. The peak around 18:00 UTC is driven by North America and coincides with (1) timing of GLOBE Observer social media posts and (2) Aqua flyovers on the East Coast of the United States. In contrast, traditional data submissions from GLOBE peak between 9:00 and 11:00 UTC. Prior to 2016 and the app, GLOBE suggested cloud observations be done at local solar noon, and many traditional GLOBE schools continue this today. The unimodal distribution for traditional GLOBE data submissions is skewed toward the school day in the Middle East. The Saudi Kingdom does not currently support use of the GLOBE Observer app but very actively submits cloud observations through traditional GLOBE channels and is consistently one of the top‐contributing countries in the region.

Photographs, while qualitative, are a valuable asset to the GLOBE database because they provide visual evidence of reported phenomena (e.g., landslides, haboobs, and *Aedes aegypti* mosquito larvae) that can be independently verified by data end users. Here the considerable benefit the GLOBE Observer app brings to the GLOBE Program is the ubiquity of built‐in cameras in modern smartphones. Table 1 summarizes the number of photo submissions. Participants have submitted 10× more photos through the GLOBE Observer app than through traditional GLOBE channels over the same time period.

Table 1 summarizes the percentage of participants who submit photos. Cloud and land cover are used for comparison here because they both include the same six directional photos (north, south, east, west, up, and down). The app uses the internal compass and gyroscope on a participant's device and automatically takes the photo when it detects the device is in the correct orientation and direction. This standardizes photos across observations. Taking photographs is optional but strongly encouraged. Missing photos does not invalidate an observation, but the inclusion of photos can increase the observation's trustworthiness and information content (e.g., land cover photos might confirm the reported presence of impervious surfaces and provide contextual clues that an area is undergoing urbanization). The app's design has evolved to encourage participants to take photos with their observation. We see evidence that the intentional design change is working when we compare the older cloud tool to the newer land cover tool. Participants submit land cover photos in all six directions at twice the rate of clouds. Design modifications to the app, such as pop‐up reminders or reward messages, could potentially close this gap.

## 4 Data Quality and Limitations

**Figure 6:**
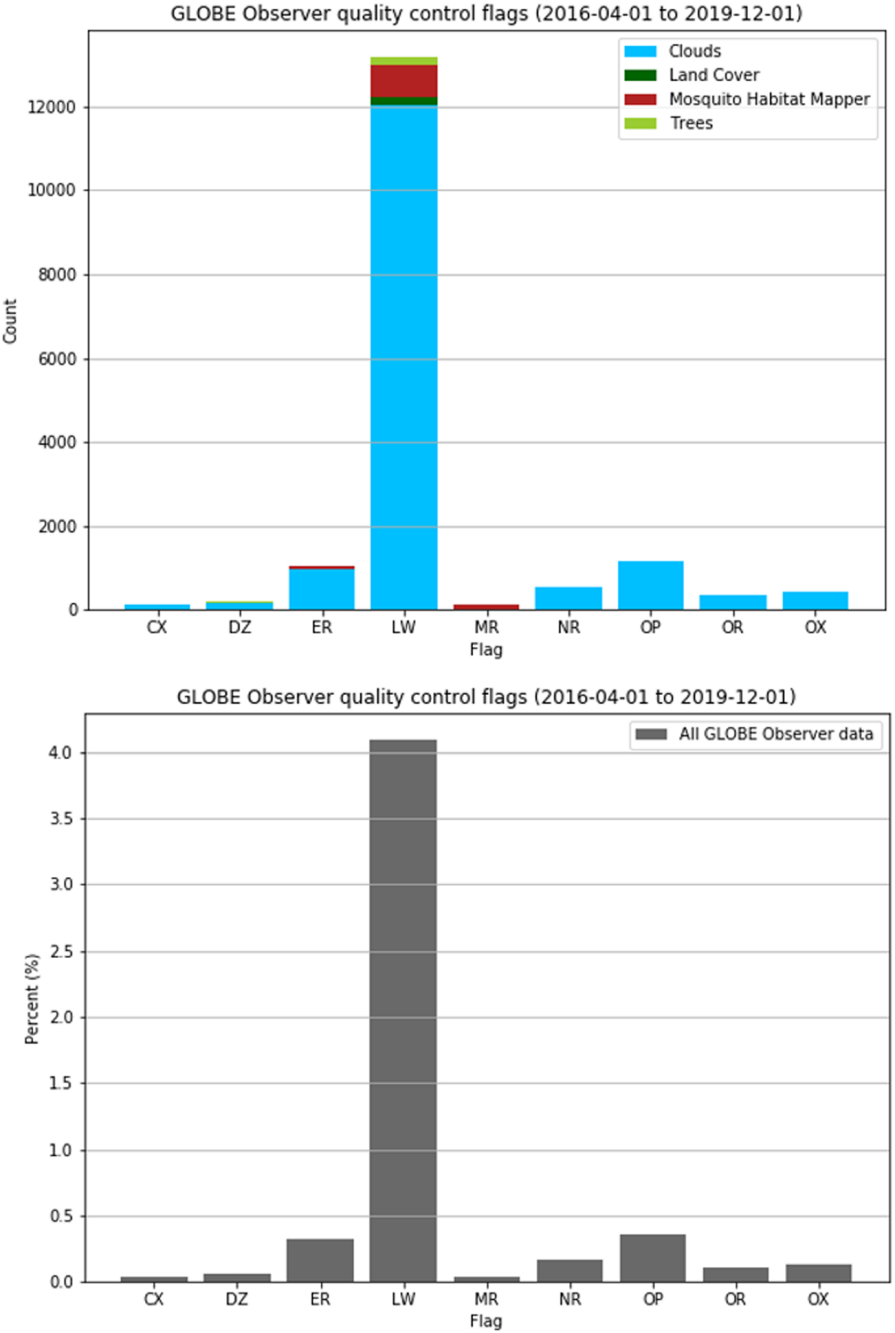
Total number (top) and percent (bottom) of flagged GLOBE Observer observations between 1 April 2016 and 1 December 2019. Flags are defined in Table 2. Here we use Cartopy's 50‐m resolution earth geometry as a land/ocean mask for the LW flag.

Figure 6 shows the percent of observations submitted through the app flagged by quality checks. The system of quality flags presented here was developed and piloted in summer 2019 and has not yet transitioned to be operational. Flag definitions and logic are in Table 2. The flags are an adaptation of Foody et al. (2017), which is an adaptation of ISO 19157 Standard Quality Measures, and of traditional GLOBE range and logic checks (GLOBE, 2019a). The suite of flags checks for logical consistency, temporal quality, and geospatial quality. An observation being flagged does not necessarily mean the observation is invalid. End users should closely scrutinize flagged observations and determine if the observation is valid for their particular application. Between April 2016 and December 2019, we find 7.8% of all GLOBE Observer app observations were flagged compared to 13% of observations submitted through traditional GLOBE channels.

**Table 2:** Quality Flag Definitions and Logic

Flag	Observation	Definition	Logic
CX	Clouds	Cloud cover is blank	Cloud cover is required
DZ	All	Datetime is exactly 00:00:00 UTC	Participant may have elected to manually record the datetime and made an error
ER	All	Elevation is outside expected range (−300, 6,000 m)	May indicate the location is erroneous
LW	All	Location may be over water	May indicate the latitude and longitude are erroneous (see section 4)
MR	Mosquito habitats	Mosquito count is outside expected range (0, 199)	Very large mosquito counts (e.g., 10,000) may be erroneous and should be used in statistical analyses with great caution
NR	Clouds	Total number of contrails is outside expected range (0, 19)	Very large contrail counts (e.g., 500) may be erroneous and should be used in statistical analysis with great caution
OP	Clouds	Sea spray reported over land	May indicate the latitude and longitude are erroneous
OR	Clouds	Three or more obscuration types reported	May indicate the participant is confused
OX	Clouds	Sky reported as obscured, but no obscuration type selected	If participant reports the sky is obscured, obscuration type should be selected

The set of flags HC, OD, OP, OR, or OX (see Table 2) checks for logical consistency around reports of haze or other obscurations in the sky. These results are consistent with anecdotal participant feedback that it is confusing in the app how to correctly report the presence of haze, smoke, and dust. The flag set MR and NR checks user‐reported mosquito larvae and contrail counts for unexpectedly large values (e.g., mosquito larvae count = 1,000,000). ER checks that the reported elevation is between 6,000 and −300 m (GLOBE, 2019a). Observations flagged ER were measured over the ocean where a negative elevation is returned reflecting the ocean bathymetry. Data consumers are advised to use caution with such flagged data in statistical analyses. For both sets of flags, modest revisions of the app's design could potentially remedy the data quality issue.

The most commonly triggered flag, “LW,” checks if an observation might be reported over water and is intended to alert data consumers to potentially erroneous locations. LW flags account for more than half of the total flag occurrences in the GLOBE Observer data set. Cloud observations are valid if taken on land or water (e.g., aboard a ship). Land cover, tree height, and mosquito habitat observations should only be collected on land. Here we use Cartopy's 50‐m resolution earth geometry for our land/ocean mask. Most of the observations flagged LW are valid upon closer inspection; they are taken on land within 50 m of a coastline or are cloud observations reported aboard a ship. A small number (<0.05%) of tree, land cover, and mosquito observations report a location over the open ocean and appear truly erroneous. This may be due to location “spoofing” by a participant to obscure their location (Zhao & Sui, 2017), or reported performance issues with the app's map function when a participant's device is out of cellular or Wi‐Fi range and cannot retrieve reliable GPS coordinates.

A limitation of GO data not captured in Figure 6 is unclassified observations. Classifying cloud type, land cover type, or mosquito genus is optional in the app. Cloud type is classified only 68% of the time, land cover type 43%, and mosquito genus only 14%. Unclassified images are still valuable because they provide a durable visual record of what a participant reports that an end user can check. However, lack of classification limits discoverability by automated keyword searches. A small pilot project is being conducted internally to explore the use of crowdsourcing to label unclassified GLOBE Observer photos to overcome this limitation.

## 5 Lessons Learned

Several lessons have been learned about participant photos. Outreach messaging coming from GLOBE Observer has predominantly aimed at encouraging participants to complete the classification steps in the app (i.e., classify cloud type, land cover type, or mosquito type). However, it is evident in the available data that a majority of participants will take photos and a much smaller percentage will complete the in‐app classification. End users of the data have expressed a strong preference for observations that include photos. A mechanism for scientists to ask participants to take photos at a particular time, location, phone orientation, or of a particular phenomenon is highly requested. Taken together, this suggests outreach messaging and in‐app user experience should pivot and optimize for targeted data collection photo‐taking. Photo labeling could be crowdsourced on a platform like Zooniverse to assemble a training data set for AI‐assisted image classification (e.g., Fortson et al., 2018; Willi et al., 2019). AI‐assisted image classification could facilitate rapid in‐app feedback to participants and would increase the information content available for research. Early work using Amazon's Rekognition™ AI software is in progress by the GLOBE Data Information Systems (DIS) team to detect and blur out human faces and text such as automobile license plates.

Cloud satellite matching is popular with participants and is being leveraged in research to a greater extent than any data product (Ault et al., 2006; Chambers et al., 2017; Colón Robles et al., 2020; Dodson et al., 2019). Satellite matching is performed by a team at NASA Langley Research Center with the goal of combining ground‐based and spaceborne perspectives to increase the amount of information about a single cloud scene. The analysis here adds to the growing body of evidence supporting the value of satellite matching. Since 2017, 203,000 observations have been matched to Aqua, Terra, CALIPSO, GOES, Himawari, and Meteosat satellite retrievals. We find here that the GLOBE Observer data set contains 2 orders of magnitude more cloud observations than any other type of observation. We also find satellite overpasses contribute to peak submission times over the course of a day. This suggests that the expansion of satellite matching to the mosquito, land cover, and tree tools in the app could be a worthwhile investment for the GLOBE Observer project team. The satellite matching component has been an effective way to engage citizen scientists and provides co‐located, independent data that can be leveraged in research.

## 6 Conclusions

GLOBE Observer is a NASA‐sponsored international citizen science project (observer.globe.gov) that is part of the GLOBE Program founded in 1995 (globe.gov). This article presents the first summary of the GLOBE Observer data set. GLOBE Observer launched a mobile app in 2016 for Android and iPhone devices that anyone in a participating GLOBE country can use to make observations (including photographs) of cloud cover and cloud type, mosquito breeding sites and mosquito type, land cover type, and tree height.

Between 1 April 2016 and 1 December 2019, 38,000 participants have submitted 290,000 cloud, 19,000 mosquito, 8,400 land cover, and 9,500 tree observations spanning all seven continents. This represents as 1.91‐fold increase in data volume in the GLOBE Program's database for these four protocols over the period of April 2016 to December 2019 and a substantial expansion in geographic coverage. The majority of observations are submitted from Europe and North America between the hours of 11:00–18:00 UTC. About half as many GLOBE Observer observations (7.8%) as traditional GLOBE observations (13%) are flagged for quality; in both cases, the most likely reason for an observation to be flagged is for the location potentially being over water. GLOBE Observer data are made publicly available for everyone (observer.globe.gov) and offer a novel ground‐based data set to augment spaceborne, airborne, and in situ Earth observations. Analysis of the data here suggests the satellite matching for clouds is a notably successful feature. The data suggest expansion of satellite matching to the land cover, mosquitoes, and tree app tools; and optimization for targeted photo‐taking could be productive avenues of development.

## Conflicts of interest

The authors have no conflicts of interest.

## Data Availability

GLOBE Observer data are publicly available (at observer.globe.gov). The Python code to read, analyze, and visualize GLOBE data for this article is available on Zenodo (https://doi.org/10.5281/zenodo.3872403).

## References

[ess2605-bib-0001] Aïkpon, R. , Dramane, G. , Klotoé, J. R. , Brettenny, M. , Lawani, Y. , Aïkpon, G. , & Yadouléton, A. (2019). Assessment of population dynamics and biting trends of *Aedes aegypti* in northern Benin: Public health implications. International Journal of Entomology Research, 4, 57–62.

[ess2605-bib-0002] AngularJS . (2015). Google, Version 1.4.3, https://angularjs.org/

[ess2605-bib-0003] Ault, T. W. , Czajkowski, K. P. , Benko, T. , Coss, J. , Struble, J. , Spongberg, A. , Templin, M. , & Gross, C. (2006). Validation of the MODIS snow product and cloud mask using student and NWS cooperative station observations in the Lower Great Lakes Region. Remote Sensing of Environment, 105, 341–353. 10.1016/j.rse.2006.07.004

[ess2605-bib-0004] Berglund, K. (1999). World Wide Weather: Involving students in GLOBE's real‐life scientific research. Science and Children, 5 10.2505/4/sc99_037_03_31

[ess2605-bib-0005] Chambers, L. H. , McKeown, M. A. , McCrea, S. A. , Martin, A. M. , Rogerson, T. M. , & Bedka, K. M. (2017). CERES S'COOL Project update: The evolution and value of a long‐running education project with a foundation in NASA Earth Science missions. Bulletin of the American Meteorological Society, 98, 473–483. 10.1175/bams-d-15-00248.1 32601503 PMC7323608

[ess2605-bib-0006] Chambers, L. H. , Young, D. F. , Costulis, P. K. , Detweiler, P. T. , Fischer, J. D. , Sepulveda, R. , Stoddard, D. B. , & Falcone, A. (2003). The CERES S'COOL Project. Bulletin of the American Meteorological Society, 84(6), 759–766. 10.1175/bams-84-6-759

[ess2605-bib-0007] Chandler, M. , See, L. , Copas, K. , Bonde, A. M. Z. , López, B. C. , Danielsen, F. , Legind, J. K. , Masinde, S. , Miller‐Rushing, A. J. , Newman, G. , Rosemartin, A. , & Turak, E. (2017). Contribution of citizen science towards international biodiversity monitoring. Biological Conservation, 213, 280–294. 10.1016/j.biocon.2016.09.004

[ess2605-bib-0008] Colón Robles, M. (2019). See a dust storm? Submit your photos with the GLOBE Observer app. *The GLOBE Program* 5 July 2019, accessed 25 February 2020, https://www.globe.gov/web/marile.colonrobles/home/blog/-/blogs/58493073

[ess2605-bib-0009] Colón Robles, M. , Amos, H. M. , Dodson, J. B. , Bouwman, J. , Rogerson, T. M. , Bombosch, A. , Farmer, L. , & Taylor, J. E. (2020). Clouds around the world: How a simple data challenge became a worldwide success. Bulletin of the American Meteorological Society. 10.1175/BAMS-D-19-0295.1

[ess2605-bib-0010] Colón Robles, M. , Farmer, L. , Dodson, J. B. , Tackett, J. L. , Cruickshank DeFontest, C. , Ivey, K. , Rogerson, T. M. , & Taylor, J. E. (2018). Citizen science cloud observations compared to near ground cloud observations from CALIPSO and MODIS satellite data over the Drake Passage. Washington, DC: American Geophysical Union Fall Meeting.

[ess2605-bib-0011] Colón Robles, M. , Rogerson, T. M. , & Dodson, J. B. (2019). NASA GLOBE Clouds: Documentation on how satellite data is collocated to ground cloud observations (v1.0). https://www.globe.gov/web/s-cool/home/satellite-comparison

[ess2605-bib-0012] Cordova, A . (2019). Adobe, version 9, https://cordova.apache.org/

[ess2605-bib-0013] Dodson, J. B. , Robles, M. C. , Taylor, J. E. , DeFontes, C. C. , & Weaver, K. L. (2019). Eclipse across America: Citizen science observations of the 21 August 2017 total solar eclipse. Journal of Applied Meteorology and Climatology, 58, 2363–2385. 10.1175/jamc-d-18-0297.1

[ess2605-bib-0014] Finarelli, M. G. (1998). GLOBE: A worldwide environmental science and education partnership [journal article]. Journal of Science Education and Technology, 7(1), 77–84. 10.1023/a:1022588216843

[ess2605-bib-0015] Foody, G. , See, L. , Fritz, S. , Mooney, P. , Olteanu‐Ramond, A.‐M. , Costa‐Fronta, O. , & Antonou, V. (2017). Mapping and the citizen sensor Mapping and the citizen sensor (Chap. 1, pp. 1–12). London: Unbiquity Press 10.5334/bbf.a

[ess2605-bib-0016] Fortson, L. , Wright, D. , Lintott, C. , & Trouille, L. (2018). Optimizing the human‐machine partnership with Zooniverse. *arXiv* Retrieved 26 February 2020, from https://ui.adsabs.harvard.edu/abs/2018arXiv180909738F

[ess2605-bib-0017] GLOBE . (2019a). GLOBE data user guide v1, GLOBE‐19‐001, NASA Goddard Space Flight Center, https://www.globe.gov/globe-data/globe-data-user-guide

[ess2605-bib-0018] GLOBE . (2019b). GLOBE Thailand engages in series of 2018–2019 trainings to ‘shut down mosquito population’ and reduce threat of mosquito‐borne disease. 29 May 2019, accessed 25 February 2020, https://tinyurl.com/v3enycq

[ess2605-bib-0019] GLOBE . (2020). Trees around the GLOBE. accessed 4 February 2020 from https://www.globe.gov/es/web/trees-around-the-globe/overview/community

[ess2605-bib-0020] GLOBE Observer . (2019). Eclipse 2019 in South America. accessed 25 February 2020, https://observer.globe.gov/en/news-events-and-people/events/obseventsdetail/19589576/eclipse-2019

[ess2605-bib-0021] Hayden, L. , Taylor, J. , & Robles, M. C. (2019). GLOBE: Connecting to community of observers directly to NASA satellites. IEEE Geoscience and Remote Sensing Magazine, 7, 98–99. 10.1109/MGRS.2019.2891930

[ess2605-bib-0022] Lee, R. B. , Priestley, K. J. , Barkstrom, B. R. , Thomas, S. , Al‐Hajjah, A. , Paden, J. , Pandey, D. K. , Wilson, R. S. , & Smith, G. L. (2000). Terra spacecraft CERES flight model 1 and 2 sensor measurement precisions: Ground‐to‐flight determinations. San Diego: Earth Observing Systems V, Proceedings Vol 4135. 10.1117/12.494216

[ess2605-bib-0023] Low, R. , Soeffing, C. , Schwerin, T. G. , Baguio, M. , Ferrell, T. , & Janney, D. (2019, 9–13 December 2019). Combining NASA GLOBE Observer with web technologies to broaden high school student access to authentic science research experiences. San Francisco: American Geophysical Union 2019 Fall Meeting.

[ess2605-bib-0024] Means, B. (1998). Melding authentic science, technology, and inquiry‐based teaching: Experiences of the GLOBE Program. Journal of Science Education and Technology, 7(1), 97–105. 10.1023/A:1022592317752

[ess2605-bib-0025] Muller, C. L. , Chapman, L. , Johnston, S. , Kidd, C. , Illingworth, S. , Foody, G. , Overeem, A. , & Leigh, R. R. (2015). Crowdsourcing for climate and atmospheric sciences: Current status and future potential. International Journal of Climatology, 35, 3185–3203. 10.1002/joc.4210

[ess2605-bib-0026] NASA . (2020). MODIS Land Cover Type/Dynamics. accessed 21 May 2020 from https://modis.gsfc.nasa.gov/data/dataprod/mod12.php

[ess2605-bib-0027] Nugent, J. (2018). Cloudy with a chance of “cirrus” science: NASA Globe Observer Clouds. Science Scope, 42(2), 26–27.

[ess2605-bib-0028] Parkinson, C. L. (2003). Aqua: An Earth‐Observing Satellite mission to examine water and other climate variables. IEEE Transactions on Geoscience and Remote Sensing, 41(2), 173–183. 10.1109/TGRS.2002.808319

[ess2605-bib-0029] Rahman, I. U. , Czajkowski, K. , Jiang, Y. , & Weaver, K. (2019). Validation of GLOBE citizen science air temperature observations using data from the Great American Solar Eclipse In Celebrating the 2017 Great American Eclipse: Lessons learned from the path of totality (Vol. 516, pp. 501–509). Orem: Astronomical Society of the Pacific.

[ess2605-bib-0030] Rock, B. N. , Blackwell, T. R. , Miller, D. , & Hardison, A. (1997). The GLOBE Program In Cohen K. C. (Ed.), Internet links for science education: Student‐scientist partnerships (Vol. 4, pp. 17–30). US: Springer 10.1007/978-1-4615-5909-2_3

[ess2605-bib-0031] Schwerin, T. G. , & Clark, A. (2019). 2019 Authentic STEM: The NASA Earth Science Education Collaborative is fostering deep engagement in NASA Earth science and research by learners of all ages. 4 November 2019, accessed 21 May 2020, http://bit.ly/NESEC_StoryMap_1

[ess2605-bib-0032] Weaver, K. , Kohl, H. , Martin, A. , & Burdick, A. (2019). How cool was the eclipse? Collecting Earth Science data with citizen scientists and GLOBE Observer In Celebrating the 2017 Great American Eclipse: Lessons learned from the path of totality (Vol. 516, pp. 511–524). Orem: Astronomical Society of the Pacific.

[ess2605-bib-0033] Willi, M. , Pitman, R. T. , Cardoso, A. W. , Locke, C. , Swanson, A. , Boyer, A. , Veldthuis, M. , & Fortson, L. (2019). Identifying animal species in camera trap images using deep learning and citizen science. Methods in Ecology and Evolution, 10, 80–91. 10.1111/2041-210x.13099

[ess2605-bib-0034] Winker, D. , Pelon, J. , & McCormick, M. P. (2003). CALIPSO mission: Spaceborne lidar for observation of aerosols and clouds (Vol. 4893). SPIE. 10.1117/12.466539

[ess2605-bib-0035] Zhao, B. , & Sui, D. Z. (2017). True lies in geospatial big data: Detecting location spoofing in social media. Annals of GIS, 23, 1–14. 10.1080/19475683.2017.1280536

